# Supplementary figures and images for: Integrated Meta-Analysis Identifies Keratin Family Genes and Associated Genes as Key Biomarkers and Therapeutic Targets in Metastatic Cutaneous Melanoma
Source: Diagnostics (Basel). 2025 Jul 13;15(14):1770. doi: 10.3390/diagnostics15141770 (PMC12293645; doi:10.3390/diagnostics15141770)

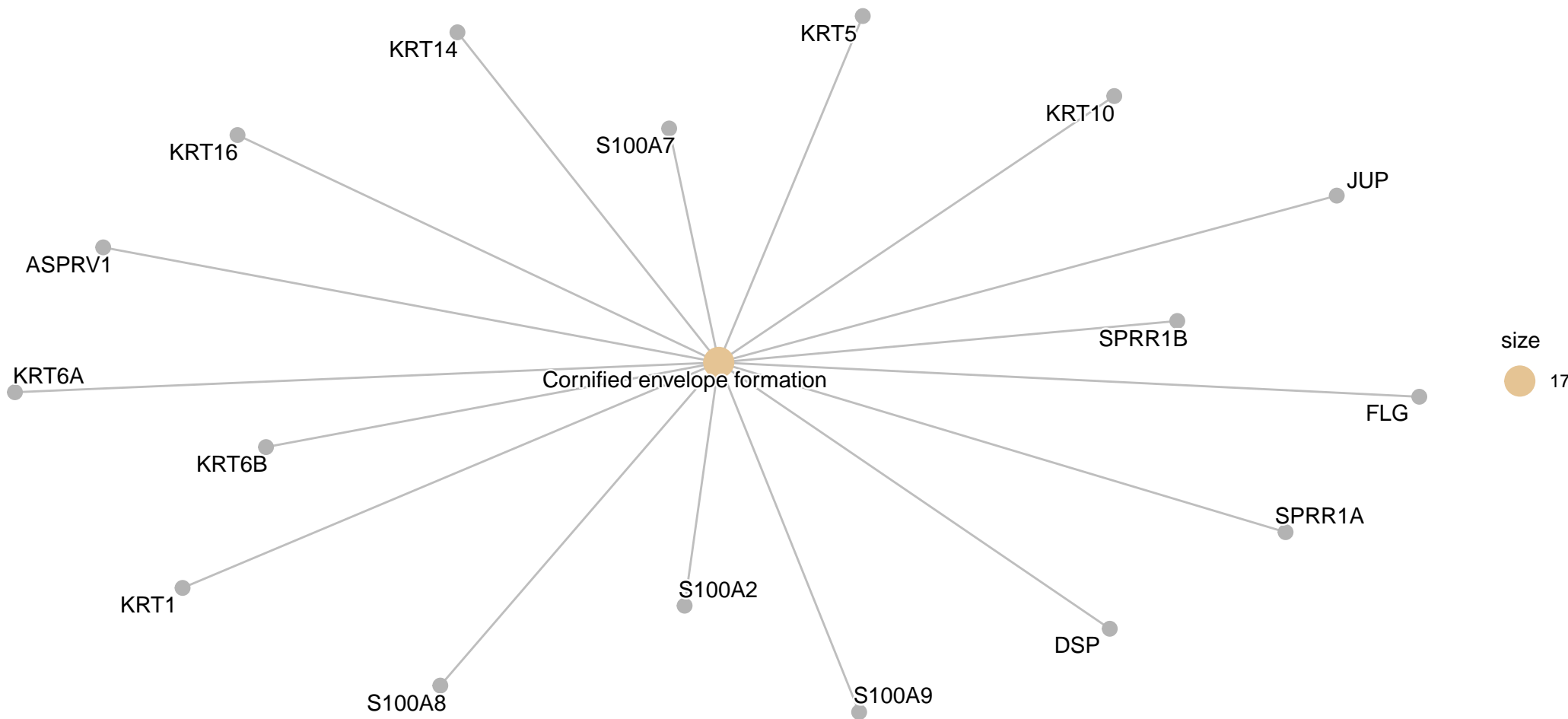

Supplement: Supplementary file 1 [file diagnostics-15-01770-s001.zip › Figure S1A_supplementary.pdf]

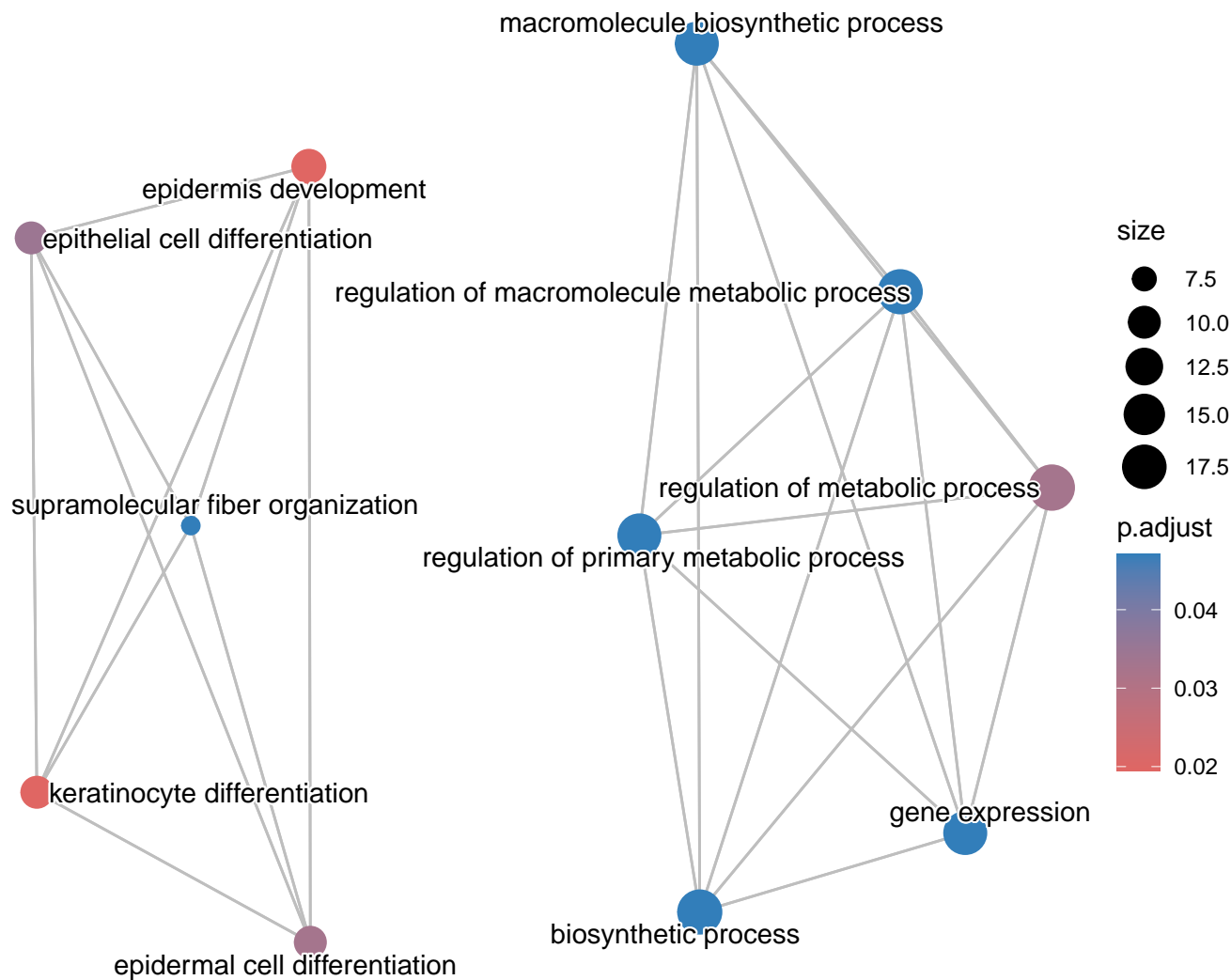

Supplement: Supplementary file 1 [file diagnostics-15-01770-s001.zip › Figure S1B_supplementary.pdf]
